# Impact of Psychiatric Hospitalization on Trust, Disclosure and Working Alliance with the Outpatient Psychiatric Provider: A Pilot Survey Study

**DOI:** 10.7759/cureus.4515

**Published:** 2019-04-22

**Authors:** Awais Aftab, Christine LaGrotta, Stephen J Zyzanski, Prakash Mishra, Syed Muhammad Ahsan Mehdi, Kathy Brown, James J Werner, Andrew W Hunt

**Affiliations:** 1 Psychiatry, University of California San Diego, San Diego, USA; 2 Psychiatry, Icahn School of Medicine at Mount Sinai, New York, USA; 3 Family Medicine and Community Health, University Hospitals Cleveland Medical Center/ Case Western Reserve University School of Medicine, Cleveland, USA; 4 Psychiatry, University Hospitals Cleveland Medical Center/ Case Western Reserve University School of Medicine, Cleveland, USA; 5 Psychiatry, University Hospitals Cleveland Medical Center, Cleveland, USA; 6 Family Medicine and Community Health, Case Western Reserve University School of Medicine, Cleveland, USA

**Keywords:** inpatient hospitalization, patient experience, working alliance, trust, disclosure

## Abstract

Introduction

The relationship between inpatient psychiatric experience and subsequent outpatient psychiatric care remains highly understudied. We conducted a voluntary, anonymous, self-report, pilot survey study to explore the impact of current or recent psychiatric hospitalization on patients’ ability to trust their outpatient psychiatric providers, particularly with respect to the disclosure of symptoms such as suicidal thoughts.

Methods

A survey was conducted in a psychiatry practice-based research network (PBRN) of six outpatient community psychiatry clinic sites within four regional agencies and at an adult inpatient psychiatry unit of a tertiary-care academic hospital in the Cleveland area. We asked patients to record characteristics of their hospitalization, perceived changes in attitudes, and complete a working alliance inventory. Sixty-two surveys were collected.

Results

Most respondents had high working alliance scores with their outpatient providers and a low prevalence of coercive experiences during hospitalization. A minority (15%) experienced a reduction in trust with their outpatient provider. Nonetheless, a substantial percentage of respondents expressed a lower likelihood of disclosing various concerning psychiatric symptoms and behaviors to their outpatient provider. Thirty-six percent reported they are less likely to disclose thoughts of harming self. Percentages for subjects reporting a reduced likelihood of disclosing thoughts of harming others, hearing voices, not taking medications as prescribed, and substance use ranged from 21-29%. At the same time, there were also trust-enhancing effects: a substantial number of patients reported an increase in their ability to trust psychiatric providers and an increase in the likelihood of disclosure of psychiatric symptoms. Exploratory analyses revealed significant associations of gender, race, outpatient provider involvement in hospitalization, and involvement of police during admission with trust, disclosure, and working alliance.

Conclusion

Even with a high therapeutic alliance and low perceived coercion during inpatient psychiatric hospitalization, the experience can lead to a disruption of trust and transparency with the outpatient psychiatrist in a considerable proportion of patients.

## Introduction

The way patients make sense of their inpatient psychiatric experience may have a bearing on how they approach subsequent psychiatric care. A review of the existing and relatively sparse literature reveals a confusing mixture of different outcomes and conflicting findings. Patient perceptions of inpatient psychiatric admissions span across a spectrum of attitudes ranging from seeing the admission as favorable-necessary-justified to unfavorable-unnecessary-unjustified, with many patients describing ambivalence [1-4]. A qualitative study by Sibitz et al. reveals the remarkable varieties of ways in which subjects integrate their experiences of involuntary hospital admission into their life narrative [1]. The authors categorized the experience of involuntary psychiatric care into three themes as a ‘necessary emergency brake’, an ‘unnecessary overreaction’, or a ‘practice in need of improvement’.

In one study, reluctance to seek outpatient treatment due to fear of coerced treatment was significantly more likely in patients with a lifetime history of involuntary hospitalization, but several other studies have found no association between experiences of coercion during inpatient psychiatric admission and a variety of subsequent outcomes [5]. Rain et al. found that perceived coercion neither increases nor decreases psychiatric inpatients' medication adherence or use of treatment services after discharge. In another report, community follow-up engagement was unaffected by involuntary hospitalization despite high correlation of perceived coercion [6-7].

Roche et al. have investigated factors associated with therapeutic relationship during psychiatric admission, and they describe that individuals who had been admitted involuntarily or who report higher levels of perceived pressures on admission are more likely to have a poorer therapeutic relationship with the consultant psychiatrist in the hospital [8]. However, the relationship between psychiatric hospitalization and therapeutic relationship with the outpatient psychiatrist remains understudied. It also remains understudied whether inpatient psychiatric experience is linked to a decreased ability to trust psychiatrists in subsequent outpatient care, particularly with respect to the disclosure of symptoms such as suicidal thoughts or psychotic symptoms.

Based on this appraisal of the existing literature, and these understudied questions, we conducted a pilot survey study to explore how patients describe their experience of a recent psychiatric hospital stay (currently inpatient or within the last six months), the perceived impact it has on their ability to trust outpatient psychiatric providers and on their ability to be forthcoming with them subsequent to the hospitalization.

## Materials and methods

This study was carried out utilizing a self-report survey questionnaire. The survey consisted of three sections.

Section one inquired about demographic information and asked questions related to the experience of psychiatric hospitalization. We also included a question utilizing summary statements of hospitalization experience in accordance with themes reported by Sibitz et al. of admission as a ‘necessary emergency brake’, an ‘unnecessary overreaction’, or a ‘practice in need of improvement’. Section two contained questions regarding an impact on the ability to trust psychiatric providers and disclose psychiatric symptoms.

Section three was a modified version of the six-item Working Alliance Scale, which is a measure of the therapeutic alliance. The six-item working alliance scale was developed and validated [9] for use during psychotherapy. For the purposes of our survey, we modified this scale for use with psychiatric providers. Our modification differs from the six-item working alliance scale for psychotherapy, which offers six responses on a Likert-type scale (not at all, a little, moderately, quite a bit, very much, completely). Our research team, in collaboration with clinical providers, felt that the modified responses would be more acceptable to our patient population. Respondents were offered six statements about the therapeutic relationship, with five possible responses on a Likert-type scale (almost never, rarely, sometimes, most of the time, almost always). Patients were asked to select the response which best described their experience with their current outpatient psychiatric provider. A working alliance score was calculated as the mean of the scores on individual responses, with a range of one to five, with one representing the lowest working alliance and five representing the highest working alliance.

For the purposes of the survey, psychiatric providers included psychiatrists as well as independent mid-level psychiatric providers (nurse practitioners and physician assistants) but excluded mental health professionals unable to provide medication management (psychologists, social workers, and case managers).

The survey questionnaire was tested for comprehension and acceptability in five patients with favorable feedback (two inpatients, three outpatients). The survey and methodology were approved by the institutional review board (approval #12-16-44).

A survey was conducted in the context of a practice-based research network (PBRN) including six outpatient community psychiatry clinic sites within four regional agencies and at an adult inpatient psychiatry unit of a tertiary-care academic hospital in the Cleveland area of Ohio, USA. Data collection was conducted in outpatient clinics from July to December 2017 and on the inpatient unit from October to December 2017.

At the outpatient sites, patients who had been admitted to an inpatient psychiatry unit within the last six months were eligible for the study. On the adult inpatient unit, all patients being discharged from the hospital were eligible for the survey. Subjects with cognitive impairment of sufficient degree to preclude comprehension of the survey were excluded from the study (however, the capacity of discernment was otherwise not formally assessed).

At the outpatient community sites, the survey could be completed by the patients in two ways: an online secure survey or a paper survey. We printed fliers for eligible subjects with information about the study and how to participate. We kept paper copies of the survey available in a marked box in patient waiting areas at outpatient clinics. Patients who wanted to fill out the paper survey could pick up the survey from the box. There was a separate drop-off box next to the pick-up box, in which patients could drop-off the filled surveys. The survey specifically stated that it was intended for subjects who had been hospitalized within the last six months, and returned surveys were subsequently screened to exclude any responses of subjects who had not been hospitalized within the specified time frame. The fliers were also given to eligible patients by their psychiatric providers. Patients were informed by their providers that the surveys are confidential and voluntary that the providers will have no knowledge of whether or how they respond, and that it would have no impact on their subsequent care.

On the inpatient unit, only paper surveys were available, since patients did not have access to the internet on the unit. The study flyer was included in the discharge packet that all patients receive during discharge preparation from the psychiatric unit. Pick-up and drop-off boxes with paper surveys were kept at the nursing counter and in the rooms where patients receive discharge instructions. A discharge nurse was available to answer any questions, but the unit psychiatrists were not involved in the survey. It was made clear to the patients that their decision to fill out the survey has no bearing on their discharge planning. The survey could only be filled out in the temporal window between receiving discharge paperwork and physically leaving the unit. 

Descriptive statistics were used for the characterization of responses. Associations between various items of the survey were examined using chi-square statistics, analysis of variance (ANOVA), and Spearman’s correlation, as appropriate. Statistical significance was set at *p-*value of 0.05. For exploratory analyses, “don’t know/not sure” answers were treated as missing values.

## Results

Survey collection

Overall, we collected 62 valid surveys, 52 from all outpatient sites, and 10 from the inpatient unit. Response rates for outpatient sites could not be calculated, since we do not know how many fliers were handed out by providers during the data collection period, and we do not know how many outpatients filled out the survey on their own without receiving a flier. One outpatient site accounted for 42.3% of outpatient survey responses. On the inpatient unit, 167 patients were discharged during the period of data collection, representing a low 6% response rate. Surprisingly, no surveys were completed online. All survey responses were completed on paper and submitted in the boxes in site waiting rooms. 

Description of survey response frequencies

The survey response frequencies are summarized in Table 1. The mean age of respondents was 41 years. There was a slight majority of females, and approximately half of the respondents were African Americans. The majority were not married. High working alliance was a pervasive and important contextual factor in the overall results. The mean working alliance score was 4.32 (SD 1.04). 

**Table 1:** Characterization of survey responses SD, standard deviation

Variables	Responses					
Survey Setting	Inpatient		Outpatient			
	10 (16%)		52 (84%)			
Age	Mean: 41.4 (SD 12.1)					
Sex	Male		Female			
	27 (44%)		34 (56%)			
Race	White		Black		Other	
	21 (35%)		32 (53%)		7 (12%)	
Marital Status	Married		Not Married			
	8 (13%)		52 (87%)			
How many times have you been admitted to a psychiatric hospital?	Once		More than Once			
	13 (21%)		48 (79%)			
When was your most recent admission?	Currently inpatient		Within last month		With last 6 months	
	10 (19%)		10 (19%)		33 (62%)	
Were you admitted against your will?	Wanted/agreed with admission		Against will			
	50 (82%)		11 (18%)			
Did your outpatient provider have a role in your admission?	Yes		No		Don’t know/Not sure	
	9 (15%)		39 (65%)		12 (20%)	
Were you pressurized into signing a voluntary form?	Yes		No		Don’t know/Not sure	
	6 (12%)		35 (73%)		7 (15%)	
Was probate court involved?	Yes		No		Don’t know/Not sure	
	2 (3%)		52 (90%)		4 (7%)	
Did you feel pressurized into taking medications?	Yes		No			
	7 (11%)		55 (89%)			
Was police involved in bringing you to the hospital?	Yes		No		Don’t know/Not sure	
	17 (28%)		37 (62%)		6 (10%)	
The admission was necessary for me to maintain safety in a time of crisis	42 (68%)					
The admission was an over-reaction, and was not helpful, and not necessary	10 (16%)					
Regardless of whether I think that being in the hospital was necessary, I am dissatisfied with the way I was treated	3 (5%)					
None of the above	12 (19%)					
How long ago did you have your first visit with your current outpatient psychiatric provider?	1^st^ visit	<6 m		6 m – 1 yr		>1 yr
	12 (20%)	12 (20%)		11 (18%)		25 (42%)
How many visits since discharge?	No previous	1 previous		2 previous		> 3
	8 (16%)	11 (22%)		7 (14%)		23 (37%)
Did your hospitalization have any impact on your ability to trust:						
Your outpatient provider	No impact		Able to trust more		Able to trust less	
	29 (49%)		21 (36%)		9 (15%)	
Psychiatrists in general	No impact		Able to trust more		Able to trust less	
	29 (49%)		22 (37%)		8 (14%)	
Since your inpatient psychiatric stay, are you more likely or less likely to report the following to your psychiatric provider:						
Thoughts of harming self	No change		More likely		Less likely	
	16 (29%)		20 (36%)		20 (36%)	
Thoughts of harming others	No change		More likely		Less likely	
	22 (41%)		18 (33%)		14 (26%)	
Hearing voices	No change		More likely		Less likely	
	25 (44%)		19 (33%)		13 (23%)	
Not taking medications as prescribed	No change		More likely		Less likely	
	22 (38%)		24 (41%)		12 (21%)	
Alcohol or recreational drug use	No change		More likely		Less likely	
	22 (40%)		17 (31%)		16 (29%)	
Working Alliance Scale overall score	Mean: 4.32 (SD 1.04)					
A. My psychiatric provider and I respect each other.	Mean: 4.55 (SD 1.05)					
B. I feel that my psychiatric provider appreciates me.	Mean: 4.23 (SD 1.20)					
C. I feel that my psychiatric provider cares about me even when I do things that he/she does not approve of.	Mean: 4.25 (SD 1.14)					
D. My psychiatric provider and I are working towards goals that we agreed on together.	Mean: 4.32 (SD 1.11)					
E. My psychiatric provider and I agree on what is important for me to work on.	Mean: 4.35 (SD 1.11)					
F. I believe the way we are working with my problem is correct.	Mean: 4.25 (SD 1.10)					

Vast majority (82%) of admissions in the survey were reportedly voluntary; almost 80% of respondents had been admitted to the psychiatric hospital more than once during their lifetime. A minority of patients reported involvement of outpatient provider in admission (15%), feeling pressurized into signing a voluntary admission form (12.5%), involvement of probate court (3%), feeling pressurized into taking medications (11%), and police involved in bringing to the hospital (28%). Majority of subjects felt that the admission was necessary for them to maintain safety in a time of crisis (67%).

Few patients indicated that there was a reduction in their ability to trust their outpatient provider (15%) and psychiatrists in general (14%). Notably, a small but nonetheless substantial percentage expressed that they were less likely to disclose concerning psychiatric symptoms and behaviors to their outpatient provider. This percentage was highest for 'thoughts of harming self' at 36%, with 26% saying less likely for thoughts of harming others, 23% for hearing voices, 21% for not taking medications as prescribed, and 29% for alcohol or recreational drug use. This disclosure-disrupting effect of psychiatric admission in a substantial subset of patients is particularly striking considering the fact that this is a population of subjects with high working alliance with their outpatient provider.

At the same time, trust-enhancing and disclosure-enhancing effects were also observed. Some subjects reported an increase in the likelihood of disclosure: 30.9% for alcohol or recreational drug use, and 41.4% to not taking medications as prescribed. More than one third of respondents reported an increase in the ability to trust their outpatient psychiatric provider and psychiatrists in general.

Exploratory analyses

We explored for associations between demographic and inpatient experience items with trust items, disclosure items, and working alliance score. Key significant findings are reported as follows. We also compared responses from inpatient subjects with outpatient subjects, but no significant differences emerged between these two groups on any of the variables.

Demographic Factors

There was a significant association between sex and impact on trust with outpatient psychiatric provider (chi square *X*^2 ^= 7.3, *p* = 0.026): compared to men, females had a greater proportion of “trust less” (26% vs 4%) and a lower proportion of “trust more” (23% vs 48%). Also, an association was also noted between race and impact on trust with outpatient psychiatric provider (*X*^2 ^= 10.3, *p *= 0.035): Blacks had a higher proportion of “trust less” (27%) responses compared to white (0%) and others (0%). Others (non-white, non-black) had a higher proportion of “trust more” (67%) responses compared to white (43%) and black (27%).

Involvement of Outpatient Provider

There was a significant association between involvement of outpatient provider in the admission and trust items: ability to trust outpatient provider (*X*^2 ^= 9.4, *p *= 0.009) and ability to trust psychiatrists in general (*X*^2 ^= 5.8, *p *= 0.054). In both cases, those whose outpatient provider was involved were more likely to report “trust more”, and those whose provider wasn’t were more likely to say “no change” or “trust less”.

Involvement of Police

Significant associations were noted between involvement of police during admission, and most of the disclosure items: thoughts of harming self (*X*^2 ^= 8.3, *p *= 0.016), thoughts of harming others (*X*^2 ^= 11.1, *p *= 0.004), not taking medications as prescribed (*X*^2 ^= 7.9, *p *= 0.019), and alcohol or recreational drug use (*X*^2 ^= 13.9, *p *= 0.001). In cases where police was involved in bringing patient to the hospital, more participants reported they were “more likely” to disclose thoughts of self-harm, harm to others, not taking medications as prescribed, and substance use. In cases where police were not involved, more participants reported being “less likely” or “no change” to disclosure on these items.

Trust Items and Working Alliance

Those who reported a reduction in their ability to trust their outpatient provider and psychiatrists in general had significantly lower working alliance scores with outpatient provider compared to those who said no impact or trust more (Table 2).

**Table 2:** Association between trust items and working alliance score N, number of subjects; SD, standard deviation

Change in Ability to Trust:	N	Working Alliance Score Mean (SD)		p-value
Outpatient Psychiatric Provider				
No Impact	29	4.47 (0.82)		0.018
Trust More	20	4.51 (0.86)		
Trust Less	9	3.43 (1.63)		
Psychiatrists in General				
No impact	28		4.49 (0.77)	0.029
Trust More	22		4.43 (1.12)	
Trust Less	8		3.42 (1.37)	

Spearman Correlations

For Spearman correlation, we assumed trust items and disclosure items to be ordinal variables arranged in the following order: trust less < no change < trust more; disclose less < no change < disclose more.

Trusting outpatient provider correlated with trusting psychiatrists in general. Disclosure items correlated with each other (Table 3): Thoughts of self-harm disclosure correlated with thoughts of harming others, hearing voices, and substance use. Thoughts of harm to others disclosure correlated with hearing voices, medication adherence, and substance use. Hearing voices correlated with substance use.

**Table 3:** Correlations between disclosure items *r, *Spearman correlation coefficient

		Thoughts of harming self	Thoughts of harming others	Hearing voices	Not taking medications as prescribed
Thoughts of harming others	r	.78			
	p-value	.001			
Hearing voices	r	.41	.31		
	p-value	002	.03		
Not taking medications as prescribed	r	.21	.37	.24	
	p-value	.13	.008	.07	
Alcohol or recreational drug use	r	.48	.39	.66	.24
	p-value	.001	.004	.001	.08

## Discussion

The results of this survey offer several insights into the outpatient impact of psychiatric hospitalization. Although the working alliance scale does not have specific cut-offs, the mean working alliance score of 4.32 (SD 1.04) is considerably high and implies that most of the patients completing the survey all shared a strong relationship with their current providers. All further results must be considered in this context. The high working alliance scores seen in our subjects are likely the result of a selection effect rather than a true representation of the targeted population: it is probable that mostly patients who had good therapeutic relationship with their psychiatric provider felt motivated to respond to the voluntary survey, and those with poor therapeutic relationship had little motivation to respond. Given this effect, our study likely represents best-case scenario responses to the effects of hospitalization. In less trusting clients, one might expect the significant outcomes of reduced disclosure to be much more likely. The low response rate from the inpatient unit may suggest that inpatients - lacking the motivation of a therapeutic relationship that outpatients had - may not have felt incentivized to respond to the survey. It should also be noted that inpatients had a short time frame available in which they could complete the survey: the few hours between receiving discharge paperwork and physically departing the unit. This short temporal window may have substantially reduced the response rate. This low response may also be a function of that specific inpatient unit which has a highly acute patient population.

Although a minority of respondents were involuntarily admitted, felt pressured into signing a voluntary form, or felt pressurized in taking medications, a substantial percentage of subjects were reportedly less likely to disclose concerning psychiatric symptoms and behaviors to outpatient providers (one third reported less likelihood of disclosing suicidal ideation). It is alarming that even with a high therapeutic alliance and low perceived coercion, the experience of inpatient admission can lead to a disruption of transparency in a considerable number of patients.

Conversely, a substantial number of patients reported an increase in their ability to trust psychiatric providers and an increase in their likelihood of disclosure of psychiatric symptoms. This would suggest that reasonable inpatient experiences were common in our sample, with a majority reporting that the admission was necessary for them in a time of crisis. However, the increase in the likelihood of disclosure may also be, at least partly, the result of a desire to avoid being re-hospitalized (i.e. disclosure of psychiatric symptoms to a trusted psychiatric provider might be calculated to reduce the chances of re-hospitalization for these subjects).

In our sample, black subjects were more likely to experience loss of trust after inpatient psychiatric hospitalization. The racial association may be related to findings from the literature that blacks are over-represented in inpatient psychiatric settings [10]. Furthermore, it has been reported that black patients may be more likely to culturally mistrust white mental health clinicians, and there are more likely to report coercion in the setting of outpatient commitment [11-12]. With regards to gender, females were more likely to experience the loss of trust in our study. Perception of being coerced into taking medications during hospital stay was associated with a low post-hospitalization working alliance. 

We noted an association between the involvement of outpatient psychiatric provider in admission and increase in ability to trust psychiatric provider and psychiatrists in general. Keeping in mind that these are subjects with a high working alliance, it appears that when the outpatient psychiatric provider is involved in the admission process patients are more likely to experience a trust-enhancing outcome of inpatient stay. This is a potentially actionable finding and would suggest that outpatients would benefit from a more active outpatient provider in the coordination of psychiatric admissions.

Police involvement in 28% of cases in our sample is consistent with mean police involvement in prior literature, which has been reported to be around 25% to 30% in the USA [13]. Involvement of police in bringing the patient to the hospital was associated with more participants in our sample reporting an increase in the likelihood of disclosure of various psychiatric symptoms. This is a relatively surprising finding; while the interaction of psychiatric patients with police has generally been assumed to be coercive in nature and police involvement has been associated with perceived coercion, it is not necessarily the case [14-15]. For instance, in a study from Canada, 72% of subjects with mental illness (not in the context of hospitalization) were satisfied with how the police had treated them [16]. It is possible that police involvement was seen in a positive manner by our survey respondents, as providing security in a time of crisis or need. Alternatively, police involvement may reflect a difficult experience, which - in combination with a high working alliance - led these individuals to be motivated not to experience re-hospitalization, and their increased disclosure to outpatient psychiatrists is a strategy to reduce risk of re-hospitalization.

**Figure 1:**
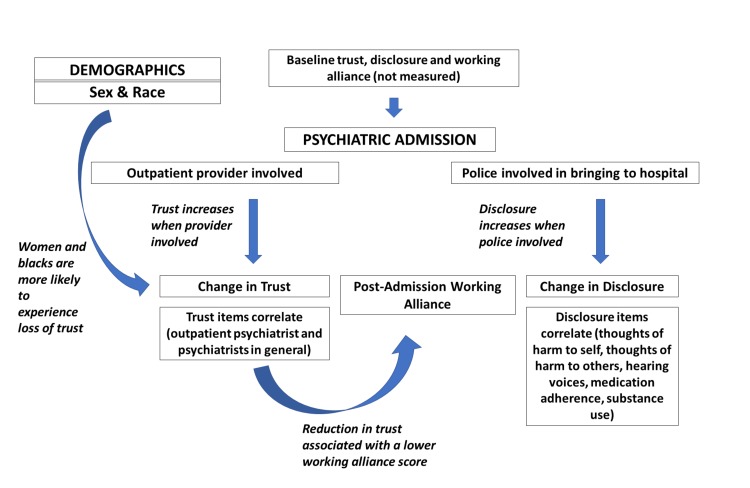
Conceptual framework

The significant findings from our analyses are summarized and integrated in a conceptual framework in Figure 1. It is our hope that this conceptual framework will help guide future researchers in this area. Future research will seek to survey larger sample sizes to validate these associations. With the proper understanding of these factors, outpatient psychiatrists may be able to develop interventions to enhance working alliance with these patients.

Our study has several strengths: the survey was conducted at multiple community sites, the data was anonymous, providers were blind to patient survey responses, assessment of items such as trust and disclosure which have been poorly studied in past literature, and incorporation of findings from prior qualitative research. There are also several limitations. Our sampling clearly excluded those with a poor working alliance, and people who do not follow-up. The sample size is relatively small and with a very low response rate from the inpatient unit. The six-item working alliance inventory was designed and validated for psychotherapy relationships, and our modified version for outpatient psychiatric use has not been formally validated. Responses regarding trust and disclosure may have been influenced by provider factors, which were not measured. The survey was purely self-report and responses may not correlate with objective behaviors. Another limitation is that psychiatric providers included practitioners with diverse backgrounds (psychiatric physicians, nurse practitioners and physician assistants). This was, however, a practical necessity as in the community psychiatry settings of North-East Ohio, these practitioners have very similar and largely overlapping roles. The fact that we did not get a single response online suggests that online surveys are likely not a good strategy for future psychiatric surveys aimed at community psychiatry populations in the North-East Ohio area.

## Conclusions

In our self-report survey study of currently or recently admitted psychiatric patients, despite the presence of high working alliance with outpatient provider and low prevalence of potentially coercive experiences, a substantial percentage expressed that they were less likely to disclose various concerning psychiatric symptoms and behaviors to their psychiatric provider after psychiatric hospitalization. Women and blacks were more likely to experience loss of trust with psychiatric providers after inpatient stay. Involvement of outpatient provider in the admission process was associated with an increase in trust, and involvement of police during admission was associated with an increase in disclosure. These findings can be integrated together in a conceptual framework. Further research is needed to understand these complex interactions, with intention to evolve supportive interventions for inpatient-outpatient transitions.
